# Intrauterine growth restriction: screening and diagnosis using animal models

**DOI:** 10.21451/1984-3143-AR2018-127

**Published:** 2020-05-22

**Authors:** Fernando Felicioni, Thaís Garcia Santos, Thaís de Mérici Domingues e Paula, Hélio Chiarini-Garcia, Fernanda Radicchi Campos Lobato de Almeida

**Affiliations:** Instituto de Ciências Biológicas, Universidade Federal de Minas Gerais, Belo Horizonte, MG, Brazil.

**Keywords:** birth weight, intrauterine environment, placenta, postnatal development, uterine crowding

## Abstract

Intrauterine growth restriction (IUGR) is a serious condition of multifactorial origin, mainly caused by maternal malnutrition, multiple gestation associated with nutrient competition, abuse of nocive substances and infections. The diagnosis of such syndrome is complex, as its own manifestations can mask its occurrence, requiring a thorough assessment of body weight and size. Moreover, it is not responsive to any kind of treatment. There is evidence that IUGR may predispose the individual to several pathologies, such as diabetes, hypertension and metabolic syndrome in adulthood, and it has also been linked to thrifty phenotype hypothesis. Thus, a healthy lifestyle is needed to better prevent those pathologies. Given the world high prevalence and importance of IUGR, mainly in developing countries, this review is focused on discussing how different animal models contribute to the biological screening and diagnosis of this condition.

## Introduction

During pregnancy, the intrauterine environment imposes the conditions in which embryo development takes place. Although genetics directly affects development, the quality of the uterine environment is a limiting factor for embryo growth and development. Genetics, therefore, safeguard organic potential while the uterine environment quality provides resources to enable the animal to express its genetic growth potential. Then, after delivery, the newborn represents the combination of these two factors acting synergistically for fetal growth during pregnancy: animal genetics and uterine environment quality (Martin-Gronert and Ozanne, 2006).

Unfortunately, insults during the prenatal life damage the quality of the uterine environment, which impairs the individual to reach its full genetic growth potential, leading to IUGR (Swanson and David, 2015; Sharma *et al*., 2016). Some examples of these insults are: uterine crowding (Alvarenga *et al*., 2013), malnutrition (Sharma *et al*., 2016), caffeine intake (Paula *et al*., 2017), alcohol ingestion (Lundsberg *et al*., 1997), tobacco smoke (Bada *et al*., 2005), cocaine use (Bada *et al*., 2005), competition for nutrients in multiple pregnancy (Puccio *et al*., 2014) and, most recently, the zika virus infection (Shi *et al*., 2018). Thus, IUGR is a result of either genetic, maternal, placental or fetal effects, as well as the combination of all these factors (Salan *et al*., 2014).

Since this syndrome is characterized by body growth restriction, a meticulous evaluation of the fetus is important to understand if the establishment of IUGR during intrauterine life is done by sparing (asymmetrical IUGR) or not (symmetrical IUGR) of the brain in growth restriction (Kurjak *et al*, 1978; Briana *et al*., 2018). The symmetric profile, originated from infection or genetic disorders, accounts for up to 30% of the cases and is characterized by a decrease in the number of somatic cells at the beginning of gestation (Sharma *et al*., 2016). This condition reflects a proportional decrease in the organs’ size and severe growth impairment in the postnatal period. On the other hand, the asymmetric profile, which represents up to 70% of the diagnoses, comes from utero-placental insufficiency (Sharma *et al*., 2016). The fetus may grow within its genetic potential in early gestation, however from 35 days on, the maternal environment will limit its normal development (Wu *et al*., 2006). The asymmetry of the organs comes from the "brain sparing effect", characterized by the redirection of the blood flow to the brain to preserve its vital functions, which compromises the development of other organs, such as the intestines and pancreas, due to low vascularization (Barbero *et al*., 2018).

In this context, the ideal measures to identify the type of growth restriction consider the association between the brain and other fetal organs. Although the ratio between brain and other organs weights is an important tool in the diagnosis of IUGR, it is feasible only in research contexts using animal models, as it requires euthanasia of pregnant dams or newborn litters. Thus, the lack of more accurate and non-invasive methods to diagnose and characterize IUGR still makes this syndrome a major challenge in both human and animal health.

Once IUGR is diagnosed, the individual will be more susceptible to other pathologies. In the last decades, it has been shown that IUGR is involved in the predisposition to pathologies that could follow up to adulthood or only be expressed at that period (Martin-Gronert and Ozanne, 2006), such as diabetes (Hales and Barker, 1992), hypertension (Hennington and Alexander, 2013) and metabolic syndrome (Jahan-Mihan *et al*., 2015), which are related to the thrifty phenotype proposed by Hales and Barker (2001). 

Those pathologies required the study of IUGR in different animal models to improve their understanding. In this regard, numerous animal species have been used under different experimental conditions. For instance, some species (e.g. mice, rat, guinea pig) are used to induce IUGR (Swanson and David, 2015), while others (i.e. humans) are used for a better understanding of the pathological consequences (Sharma *et al*., 2016). Interestingly, one species stands out for the occurrence of uninduced IUGR: the pig (Wu *et al*., 2006).

Since IUGR is the result of poor intrauterine development, it is important to understand the particularities of gestation for a better comprehension of the occurrence of this syndrome. It is well known that during the embryonic period, the organic systems are formed for further growth and maturation at the fetal period. Although the processes that occur during the embryonic period are critical for further development, nutrient demands from the mother are low in comparison to the fetal period, when occurs an intense fetal growth (King, 2000). Besides that, the mammalian eutherian species presents a key organ to help these maternal-fetal exchanges: the placenta. This organ appears to supply the increasing fetal nutritional demands during pregnancy and, during its formation, can perceive the maternal nutritional status to decide how much it will grow (Díaz *et al*., 2014). In this sense, poor maternal conditions will imply in smaller placentae and consequently poor fetal nutrition, contributing to IUGR.

Due to these gestational particularities, maternal poor nutrition, exogenous stimuli or even the natural uterine crowding, observed in pigs, have caused severe effects in prenatal development during the fetal period. Hence, IUGR has been frequently named as fetal growth restriction (FGR), characterizing the period in which it is established. In this context, this review seeks to clarify how different animal models contribute to the biological screening and diagnosis of intrauterine growth restriction.

## The murine as a model: IUGR caused by harmful maternal habits

During pregnancy, women’s health deserves special attention, seeking not only their wellbeing, but also their baby’s health status. Unfortunately, due to social and economic reasons or even misinformation, this fact is ignored, and severe consequences can compromise pregnancy, leading to IUGR.

### Low protein diet

Nutrition during pregnancy can be compromised if the diet is inadequate either in quantity or quality, which will have a negative effect on fetal development due to high nutrient demands for its body building. In this context, the experimental manipulation of diets and the variety of feeding regimens had generated evidences which suggest the metabolic syndrome as a converging phenotype (Armitage *et al*., 2004). Thus, the large amount of reported scientific evidences had shown that diet manipulation is a well-defined model to study fetal programming.

In this regard, the lack of protein is an extremely serious condition, once proteins are essential structural components of the organic systems. The consequences of this aggressive condition have been demonstrated by low birth weight in newborn pups whose mothers were submitted to low protein diets during pregnancy (Gheorghe *et al*., 2009). It has also been shown a decrease of lung alveoli in fetuses as a result of altered VEGF signaling (Liu et al., 2014).

Those evidences brought up new insights which instigated the need to investigate the causes and how they triggered those damages to the offspring. In this scenario, an organ stands out as a key player for fetal development: the placenta. It is composed of distinct layers, each one with a specific function: endocrine and metabolic (junctional zone) or maternal-fetal exchanges (labyrinthtrophoblast layer) (Isaac *et al*., 2014).

It has been shown that protein restriction during pregnancy in female rats resulted in reduced junctional zone and labyrinthtrophoblast weight when evaluated at 14 days of gestation (Gao *et al*., 2013). In a study with mice, Rutland *et al*. (2007) demonstrated that mice undergoing protein malnutrition (9% of crude protein) during gestation presented a reduction in the length of the labyrinth blood vessels and reduced fetal weight at embryonic stage (E) 18.5, suggesting that malnutrition would cause vascular dysfunction in the murine placenta. At 18 days of pregnancy in rats, it was demonstrated that protein restriction impaired the differentiation of the trophoblast, once there was an increased expression of the trophoblastic stem cell genes, Esrrb, Id1 and Id2, in the placental junctional zone (Gao *et al*., 2013).

Although studies using low protein diets exclusively during pregnancy are of great importance to understand the consequences in this context, our research group has been investigating feeding strategies which are closer to the human reality. As so, unlike strategies widely employed with the use of low protein diets from the beginning of pregnancy (e.g. short term), our group evaluated an innovative method: the consequences to pregnancy after a low protein consumption during a chronic (e.g., long term) period (Muñoz and Bongiorni-Malavé, 1979; Wainwright and Stefanescu, 1983; Ito *et al*., 2011). Considering this strategy, offspring from dams submitted to chronic low protein diet during pregnancy and lactation showed compromised postnatal development and the histomorphometrical analyses of liver, kidneys and ovaries showed alterations of those organs’ parenchyma, reflecting physiological impairment (Almeida *et al*., 2012). Recently, Gonzalez *et al*. (2016) submitted isogenic mice to chronic low protein intake and by evaluating the final stages of pregnancy (E17.5 and E18.5), it was suggested that nutrients are preferentially allocated to sustain fetal and brain growth, and the placenta may have a nutrient sensor in early gestation with a role in mitigating impacts of poor maternal nutrition on fetal growth. 

Our research group investigates the placenta as a key organ to better understand the origins of IUGR under chronic protein malnutrition, however, using a heterogenic mice model. According to recent findings, even though low protein intake affects placental and fetal growth, the prospects involve the demonstration of autophagy as an important phenomenon for fetal growth (unpublished data).

### Caffeine intake

Caffeine is an alkaloid from the trimethylxanthine group. In humans, its half-life may vary from 2 to 4.5 hours, reaching up to 12 hours. Once ingested, it is metabolized by a family of hepatic enzymes named cytochrome P450-oxygenases (CYP1A2) (Butt *et al*, 2011). Both caffeine and its metabolites (paraxantine, theobromine and theophylline) are very small and highly lipophilic molecules, having the ability to diffuse through biological tissues by passive diffusion, crossing tissues with high selective permeability, such as the placental membrane (Mose *et al*., 2008; Christian, 2001). 

During pregnancy, there is a reduction in the clearance and excretion of caffeine and its metabolites leading to a high deposition in maternal and fetal tissues (Yadegari, 2016; Jahanfar, 2015). These substances freely cross the placental membrane and, once in the placenta, the fetuses and, later the newborn, are unable to metabolize them, as their elimination is totally dependent on the renal system, which is not fully developed at that stage (Klebanoff, 2002). Recently, studies have pointed out caffeine intake during pregnancy as a causative agent of IUGR in rodents (Paula *et al*., 2017).

Huang *et al*. (2012) reported reproductive alterations in female mice treated with caffeine. All treated animals showed low conception rate and decreased maternal body weight gain during pregnancy. Moreover, an increased IUGR ratio, which was dose-dependent, was also observed. Yadegari *et al*. (2016), using intraperitoneal doses of 150 mg/kg/day of caffeine in female albino rats during 1 to 5 GD (gestational days), reported that the treated group showed a significant decrease in implantation sites and the number of live newborn, suggesting that caffeine is likely to cause anti-fertility effects. 

Momoi *et al*. (2008) reported that modest daily maternal caffeine exposure altered regional embryonic arterial blood flow development and induced IUGR. Huang *e al*. (2012) reported similar results with rats treated during pregnancy: occurrence of IUGR, decreased placental and fetal weights and decreased fetal length.

Recently, our research group evaluated biometric outcomes of caffeine in mouse placentae and fetuses at late pregnancy (GD 17.5). It was demonstrated that caffeine doses can negatively affect fetal and placental development in late pregnancy even in lower doses. Additionally, it was observed the occurrence of IUGR after oral administration of caffeine during pregnancy (data not published). In our study, female Swiss mice ingested, before and during pregnancy, 0, 60, 120 or 240 mg/kg/caffeine/day. These amounts are equivalent to 0, 100, 200 and 300 mg/caffeine/day in humans, according to the correction factor established by Wang (2005). It is important to note that the Food and Drug Administration (FDA, 2002) and the European Food Standards Agency (EFSA, 2015) suggested in the year 2000 the consumption of 200 to 300 mg per day. So, for a pregnant woman of 65 kg, these are “safe” doses. 

Much has been already concluded upon the effect of caffeine in animals and the occurrence of IUGR, although the mechanisms involved in those findings still remain unclear. More studies are needed to explain what are the structural and morphofunctional changes caused by caffeine during placental and fetal development.

Huang *et al*. (2012), by inducing a dose-dependent consumption of 180 mg/kg of caffeine during pregnancy in Wistar rats, observed edematous regions in the endoplasmic reticulum, expansion of the cisterns of the rough endoplasmic reticulum, ribosomes degranulation and heterochromatin condensation in trophoblast cells. Ma *et al*. (2015) observed an increased apoptosis and inhibitions of angiogenesis following exposure to a “chicken functional placenta” by excessive of caffeine intake. In this regard, our study showed commitment of placental vascularization due to caffeine intake (data not published). However, more studies are necessary to conclude the exact mechanism of such effects.

## The pig as a model: lessons from the pig whose IUGR is natural

Although laboratory animals have been extensively used as a model to study IUGR, phylogenetic and metabolic differences may compromise the transposition of the results to the human physiology (Óvilo *et al*., 2014). In this sense, some large animals, like pigs, are considered ideal model for extrapolating the results to humans, due to similarities in anatomy, metabolism and behavior. The feasibility of using the pig as a model for the study of IUGR can be highlighted by the spontaneous and severe occurrence in this species (Etuk, 2010). Since the last decade, the rate of IUGR in piglets has increased significantly, from 6% up to 30% of newborn. This fact coincided with the pressure of studies of genetic improvement focused on the development of females with high ovulation rates, which resulted in an increase in the number of piglets per litter (Ferenc *et al*., 2014).

The diagnosis of IUGR is complex, especially in domestic animals, and there is not yet a consensus defined. To be considered an IUGR carrier, a piglet must satisfy some criteria determined by the researcher, so that the chance of mistake is less, since there is not a biological marker. One of the most obvious parameters of IUGR is low birthweight; however, this insulated criterion is not always reliable for a diagnosis (Giabicani and Pham, 2018). 

To establish the weight range that represents IUGR and normal weight piglets, it is convenient to acquire the birth weight of approximately 1,000 piglets from the farm, originating from the same maternal genotype. In this way, it is possible to calculate the mean (μ) and the standard deviation (σ) of the variable birth weight. The weight ranges of each group are determined by a range using μ + 1 σ to μ + 2 σ for the normal weight score and μ - 2σ to μ -1σ for the IUGR score.

Many factors, such as maternal, litter of origin and anthropometric measurements of the newborn, can indicate whether or not the low birth weight comes from the restriction of fetal growth. The most important maternal factor is parity order, and in commercial farms, the dams remain in the breeding herd until the ninth parity. The first and last pregnancies should be disregarded in the criteria for sampling piglets. Primiparous sows give birth to piglets of lower birth weight than multiparous sows, because of uterine immaturity; in contrast, from the seventh parity the ovulation rate decreases drastically, which may explain the occurrence of smaller litters. The ovulation rate peak occurs between the fourth and sixth parturition, and from the 35th gestational day, uterine capacity becomes a limiting factor for fetal growth. Hence, this scenario may predict the occurrence of IUGR due to uterine and placental insufficiency and, therefore, piglets from these deliveries can be select for the trial.

Among the selection criteria for IUGR piglets, is important to establish the size of the litter, as this ensures that they remain submitted to a similar uterine environment. However, determining this number seems difficult. For example, the litter size established by Alvarenga *et al*. (2013) was based on the average size of the genotypes at that time, which was around 10 to 15 piglets. Interestingly, the current genotypes generate a greater number of individuals, which makes it hard to find litters of 10 piglets. A current study done by our research group established litter sizes between 15 to 22 piglets as selection criteria. 

As in other animal models, it is possible to measure the relationship between brain and liver weight to determine the occurrence of IUGR. This ratio reflects the brain sparing effect, and in this case, IUGR piglets clearly show an exacerbated cranial circumference compared to animals considered normal. Similarly, the developmental impairment of the liver and other organs affects the abdominal circumference, which is smaller in IUGR animals. Hence, the observation of anthropometric measures are important in the diagnosis of IUGR, especially when it is not possible to euthanize the animal.

In the fetal programming context, these anthropometric measures that involve the development of organs depend greatly on the composition of nutrients that will be transferred through the placenta (Zabielski *et al*., 2008). In piglets, insults in the intrauterine life are known to be responsible for perinatal mortality, lower postnatal growth, and compromised carcass characteristics, which leads to large economic losses to the swine industry (Alvarenga *et al*., 2013). The low body weight of an IUGR piglet remains lifelong and will hardly be corrected by compensatory weight gain. It is believed that one of the major causes of such impairment are damages to the small intestine, although it remains unclear whether these alterations persist throughout adult life (Skrzypek *et al*., 2018).

The intestinal epithelium develops strongly during postnatal life through intense cell remodeling (Zabielski et al., 2008). In the first weeks of life, for example, enterocytes have vacuoles that absorb macromolecules from colostrum and milk and disappear overtime. It is clear that in IUGR piglets, the maturation of the enterocytes is late, indicating commitment of normal digestive functions (Wang *et al*., 2005; Ferenc *et al*., 2017). This fact is still associated with the smaller weight and length of the small intestine, with reduction of the absorptive area, expressed by the lower villi / crypt ratio and microvillus lesions. The enzymatic activity of trypsin and lipase is still compromised, coupled with lower expression of IGF-1 and its receptor in the intestinal mucosa (Wang, 2005).

The cellular mechanisms of metabolic programming are still unclear. However, reprogramming of proteins responsible for cell proliferation and apoptosis, and important morphophysiological changes in systems and tissues may be involved. It should be emphasized that light piglets at birth require more days to reach the ideal weight at slaughter and require greater care in the postnatal period, leading to an increase in production costs (Ashworth, 2013). In this sense, understanding the consequences of fetal growth restriction on the gastrointestinal system in pigs can enable them to express their full growth potential, and reduce their mortality rate, increasing the economic gains.

## Final considerations

**Figure 1:**
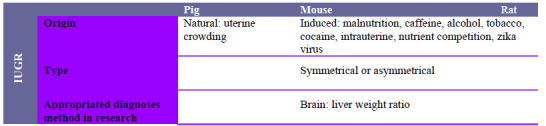
Summary of origin, types and diagnosis of intrauterine growth restriction (IUGR) in pig, mouse and rat models.

Given the scientific evidences reported herein, it can be stated that IUGR is a syndrome of multifactorial origins, and its occurrence, precise diagnosis and consequences are still a great challenge, as so far there is no treatment (Fig. 1). Therefore, the importance of different animal models is evident to potentiate the biological screening and the diagnosis precocity. Although the IUGR biological scenario is an arduous and aggressive challenge, it is undeniable that, as a public health problem, public policies are needed to combat the agents that stimulate this syndrome: hunger, malnutrition, drugs, lack of sanitation as well as clarifying the population about the consumption of inadequate substances with unrecognized biological hazard to the fetus.
